# Genetic characterization and implications for conservation of the last autochthonous Mouflon population in Europe

**DOI:** 10.1038/s41598-021-94134-3

**Published:** 2021-07-19

**Authors:** Valentina Satta, Paolo Mereu, Mario Barbato, Monica Pirastru, Giovanni Bassu, Laura Manca, Salvatore Naitana, Giovanni Giuseppe Leoni

**Affiliations:** 1 Department of Veterinary Medicine, University of Sassari, Via Vienna 2, 07100 Sassari, Italy; 2 Department of Biomedical Sciences, University of Sassari, Viale San Pietro 43b, 07100 Sassari, Italy; 3 Department of Animal Science, Food and Technology–DIANA, Università Cattolica del Sacro Cuore, Piacenza, Italy; 4 Agenzia FoReSTAS, Via Merello 86, Cagliari, Italy

**Keywords:** Population genetics, Bayesian inference, Haplotypes, Phylogeny

## Abstract

Population genetic studies provide accurate information on population structure, connectivity, and hybridization. These are key elements to identify units for conservation and define wildlife management strategies aimed to maintain and restore biodiversity. The Mediterranean island of Sardinia hosts one of the last autochthonous mouflon populations, descending from the wild Neolithic ancestor. The first mouflon arrived in Sardinia ~ 7000 years ago and thrived across the island until the twentieth century, when anthropogenic factors led to population fragmentation. We analysed the three main allopatric Sardinian mouflon sub-populations, namely: the native sub-populations of Montes Forest and Mount Tonneri, and the reintroduced sub-population of Mount Lerno. We investigated the spatial genetic structure of the Sardinian mouflon based on the parallel analysis of 14 highly polymorphic microsatellite loci and mitochondrial D-loop sequences. The Montes Forest sub-population was found to harbour the ancestral haplotype in the phylogeny of European mouflon. We detected high levels of relatedness in all the sub-populations and a mitochondrial signature of hybridization between the Mount Lerno sub-population and domestic sheep. Our findings provide useful insights to protect such an invaluable genetic heritage from the risk of genetic depletion by promoting controlled inter-population exchange and drawing informed repopulation plans sourcing from genetically pure mouflon stocks.

## Introduction

Ovine domestication began in the Fertile Crescent around 10,000 years ago (YA) from mouflon-like animals with horns and short-shedding wool^1–3^. A second wave of domestication occurred 6000 years later and led to the modern sheep: polled with no-shedding wool^4,5^. As a consequence, the rearing of the first mouflon-like ovine was abandoned in favour of the new economically valuable one^6^. The original mouflon-like went back to feral life but gradually disappeared in mainland Europe due to intense hunting and habitat erosion. However, relics of these ancestral populations survived in the harshest mountainous areas of the Mediterranean islands of Sardinia, Corsica, and Cyprus, where they established populations which survived until now^4,5,7,8^. Since the eighteenth century, Corsican and Sardinian mouflon have been used to repopulate several regions of mainland Europe. During the last century, the Corsican and Sardinian mouflon populations experienced severe size contractions due to over-hunting and habitat erosion. However, the implementation of conservation policies in the last 50 years brought the populations to increase and reach the current number of ~1000 and ~6000 individuals in Corsica and Sardinia, respectively^9–11^.

The most credited hypothesis dated the arrival of mouflon in Sardinia around 7000 YA^7,8,12,13^, followed ~3–4000 years later by the ovines of the second wave of domestication. In the twentieth century the competition for grazing land between mouflon and domestic sheep, the intensified hunting pressure, and the more recent use of barbed-wire fences along the roads strongly altered the mouflon natural habitat, resulting in population fragmentation into small, isolated groups often confined to less favourable habitats^14^. Finally, the outbreak of newly introduced infectious diseases (e.g., the blue tongue) contributed to further reduce the mouflon population size^15^.

**Figure 1:**
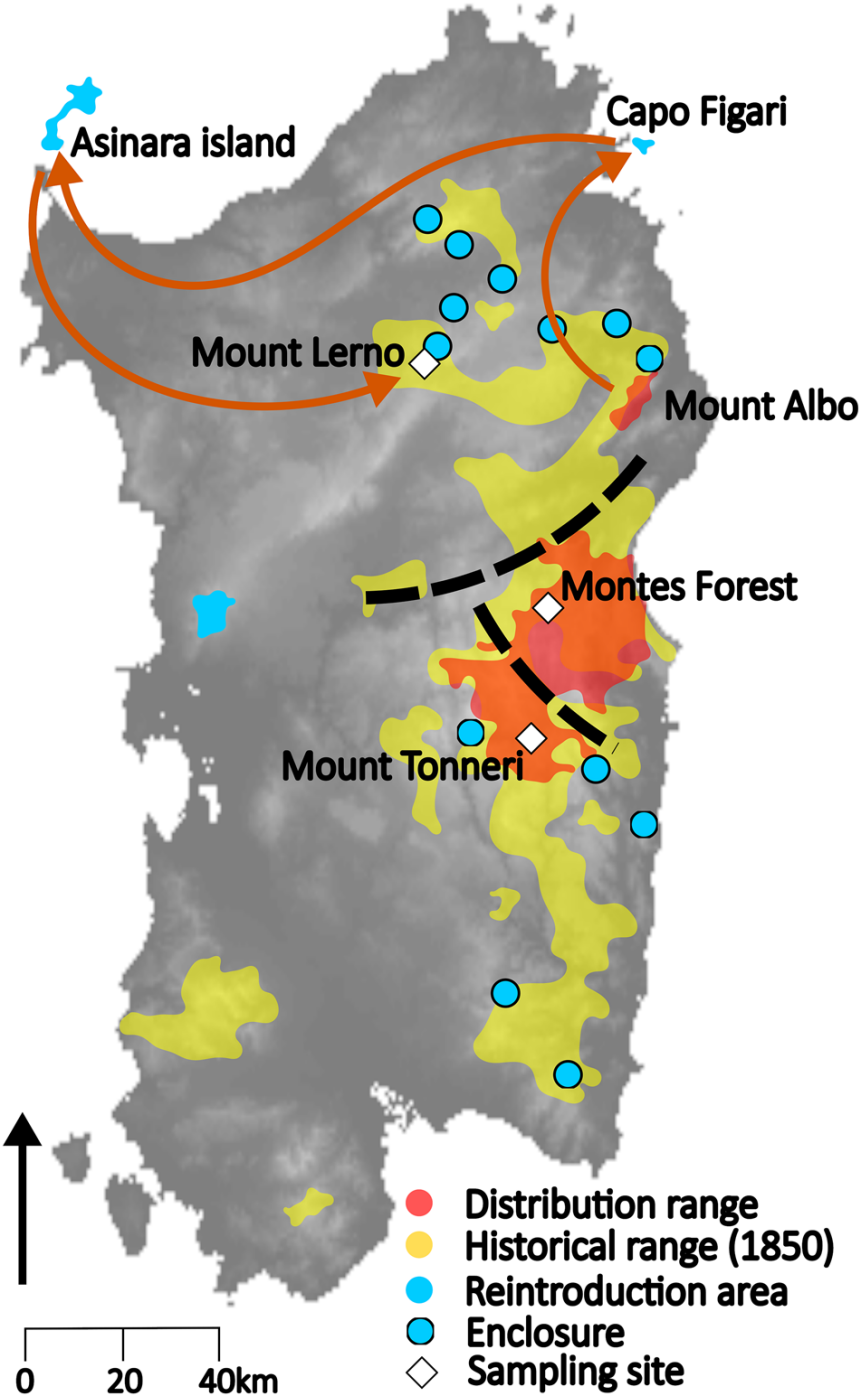
Historical and current distribution of the mouflon in Sardinia and sampling sites. Black dashed lines represent the two main regional connection roads. Red arrows indicate the three steps of translocation of the animals selected for reintroduction in the Mount Lerno area. Map was generated in Inkscape v1.0 (https://inkscape.org/).

Currently, the mouflon distribution is limited to eastern Sardinia (Ogliastra, Gennargentu and Supramonte), including the areas of Mount Tonneri (Seui), Montes Forest (Orgosolo), and Mount Albo (Lula), and a few managed and protected areas where mouflon has been reintroduced. This range covers only a limited part of the original species distribution^16^ (Fig. 1). In 1998, the Sardinian Regional Council for Environment started an action plan to increase the number and distribution of mouflon across Sardinia by transferring small groups of mouflons sourced from the historical mouflon range into enclosures before being released into new areas (Legge Regionale n 26). Population bottlenecks across several generations can lead small, isolated populations to rapidly lose genetic diversity as a result of genetic drift^17–19^, at a speed that depends on the severity of the bottleneck and the standing diversity of the founder population^20–22^. Further, in the case of the Sardinian mouflon, hybridization with domestic sheep may affect the genetic integrity of mouflon through the introgression of maladapted genetic components^7^. Crossbreeding between mouflon and sheep has been reported since Roman times^23^ and still occurs^24,25^. This is often the case for mouflon colonies established for hunting purposes in several European countries, where the practice of human-mediated crossbreeding was used to produce hybrids with increased body size and bigger horns^26^.

A better knowledge of the genetic differentiation of wildlife populations and the improved understanding of the selective pressure acting upon them are essential tools for the effective management and conservation of endangered species. Molecular markers like mitochondrial DNA (mtDNA) and microsatellites are commonly used to evaluate the genetic health of a population and to define conservation units representing valuable reservoir of genetic variation. Studies based on genome-wide markers and whole mtDNA sequence have shown low levels of genetic diversity and the occasional occurrence of domestic sheep introgression signals in the Sardinian mouflon populations^5,7,27^.

To date, five domestic sheep mitochondrial lineages, defined as haplogroups (HPGs) A, B, C, D, and E, have been identified^28^. HPG-B is the most common haplogroup among sheep breeds^28,29^ and is also shared by the mouflon from Sardinia and Corsica. The Sardinian and Corsican mouflon are classified as European mouflon (*Ovis gmelini musimon*) along with the reintroduced mouflon populations from mainland Europe, despite the great genetic distance detected between the Corsico-Sardinian and the European mouflon mtDNA lineages^5,8^. The Corsico-Sardinian mouflon clade shows an early split from the evolutionary branch originating the domestic sheep HPG-B and the mainland Europe mouflon lineage^5^. Further, the Sardinian mouflon gene pool harbours the oldest HPG-B haplotype identified so far^8^. Such findings suggest the need to revise the systematic classification and nomenclature of these species. Until a new nomenclature is established, in this report we choose to identify the individuals from mainland Europe as European mouflon, whereas we will refer to insular individuals as Sardinian or Corsican mouflon, according to their origin.

Here, we use mtDNA and microsatellite markers to describe the genetic diversity and population structure of the Sardinian mouflon. We estimate the genetic differentiation among and within three representative sub-populations from the historical range of Sardinian mouflon and evaluate the effect of landscape anthropogenic alterations. Finally, we provide the first data on the spatial genetic structure of mouflon in Sardinia.

## Results

### Mitochondrial DNA

We generated a total of 54 sequences of heterogeneous length ranging from 460 to 1180 bp which were deposited in GenBank (GB# MW727287-340). In order to estimate the genetic variability on the entire available sample, the analyses were carried out on a 439 bp fragment of the hypervariable domain I of the mitochondrial D-loop region. A total of five haplotypes were detected: Hpt-1 (GB# MG489885) previously identified in Sardinia^8^ and belonging to the HPG-B, Hpts 2–4 described for the first time in this work, and Hpt-5 (GB# KF228720) previously found in Comisana sheep^30^.

Within the Montes Forest sub-population three haplotypes separated by one-step mutations were observed, resulting in a haplotype diversity of 0.62 (Table 1). Only one private haplotype was found for each of the other two sub-populations. The five haplotypes were separated by seven polymorphic sites and represented an overall haplotype diversity of 0.74 and nucleotide diversity of 0.006 (Table 1).

**Table 1:** mtDNA D-loop haplotypes detected in Sardinian mouflon population.

Sampling area	Haplotype	N	S	h	Hd	π
Montes Forest	Hpt-1	5	2	3	0.621	0.00186
	Hpt-2	10				
	Hpt-3	3				
Mount Tonneri	Hpt-4	21	0	1	–	–
Mount Lerno	Hpt-5	15	0	1	–	–
Total		54	7	5	0.739	0.00594

Sample distribution and genetic diversity indexes sorted by sampling area.

*N* sample sizes, *S* number of polymorphic sites, *h* number of haplotypes, *Hd* haplotype diversity, *π* nucleotide diversity.

The components of the total genetic variation were estimated by AMOVA and showed a higher proportion of genetic diversity variation “among populations” (79.3%) than “within population” (20.7%) (Table 2).

**Table 2:** Results from Analysis of Molecular Variance (AMOVA) testing sub-populations of Sardinian mouflon based on partial sequences (439 bp) of mtDNA D-loop and 13 microsatellite loci.

Source of variation	df	Sum of squares	Variance components	Percentage of variation
**mtDNA (F**_**ST**_** = 0.79; *P* = 0.000)**				
Among populations	2	14.31	Va = 0.40	79.26
Within populations	51	5.28	Vb = 0.10	20.74
Total	53	19.59	0.50	100.00
**Microsatellite loci (F**_**ST**_** = 0.30; *P* = 0.000)**				
Among populations	2	95.91	Va = 1.26	29.57
Among individuals within populations	51	154.45	Vb = 0.03	0.66
Within individuals	53	151.50	Vc = 2.97	69.77
Total	106	410.86	4.26	100.00

*df* degrees of freedom.

**Figure 2:**
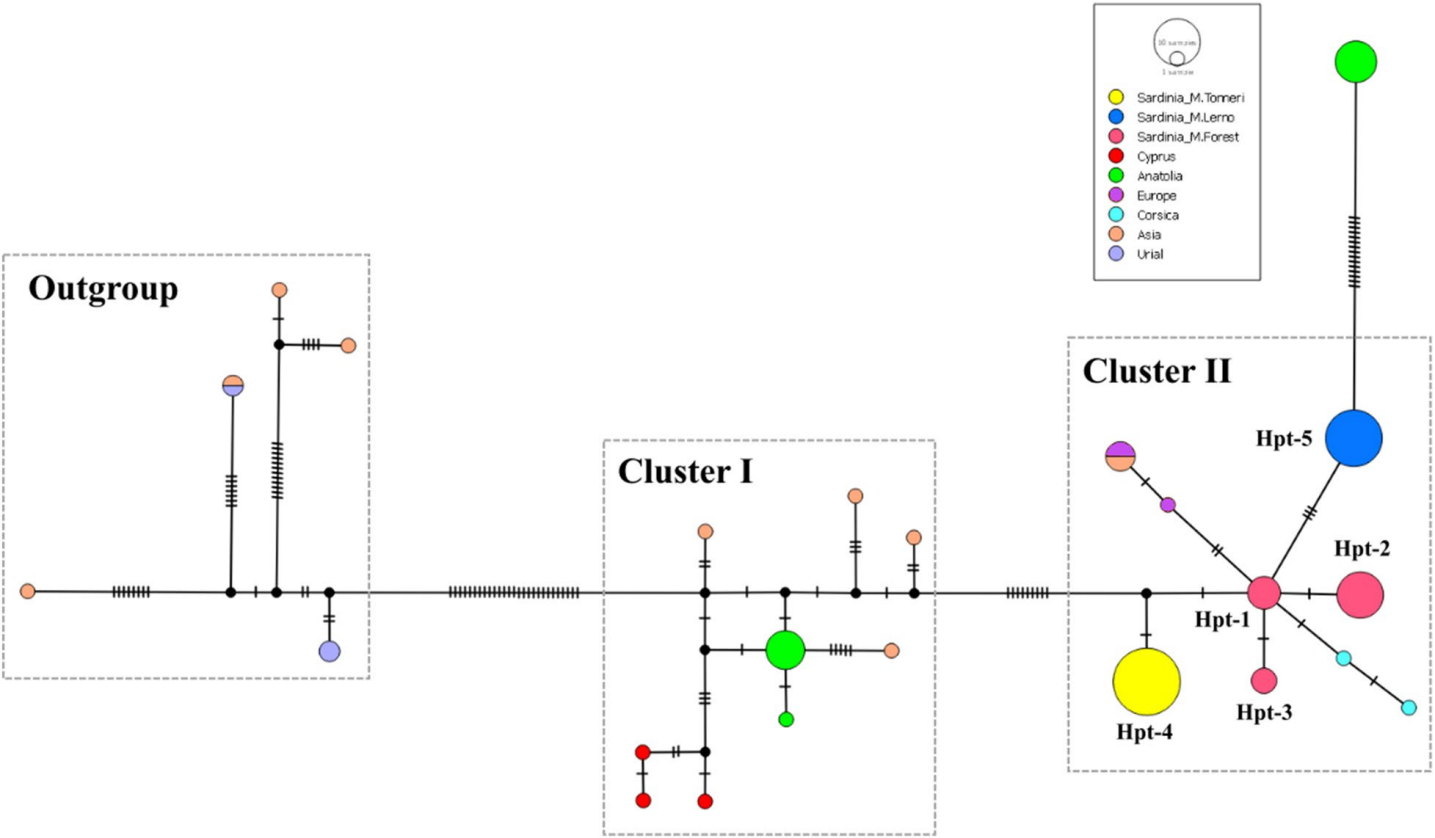
Median-joining network of mtDNA D-loop haplotypes as inferred from the analysis of 88 *Ovis gmelini* sequences. Three additional homologous sequences from urial (*O. vignei*) were included as outgroups. Haplotype clusters (Outgroups—Cluster I–Cluster II) are indicated by dashed squares. The number of mutations between haplotypes is indicated by perpendicular lines. The size of the circles is proportional to the frequency of a certain haplotype in the total sample, with colours assigned by geographical origin. Labels referring to haplotypes detected within Sardinian samples are also reported.

The median-joining network (MJN) analysis detected two main D-loop haplotype clusters well separated from the outgroup (Fig. 2). A clear geography-based cluster composition was revealed: Cluster I included sequences of mouflons from Asia and Cyprus, while Cluster II comprised all the sequences from Europe and a few from Asia. The five haplotypes found in Sardinia are represented in Cluster II. The Hpt-1 was positioned in the centre and directly connected to all the other haplotypes.

To quantify D-loop divergence within the Sardinian mouflon population (n = 54), pairwise genetic distances between groups were calculated (Supplementary Table S1). The mean divergence was 0.005 ± 0.002 and 0.01 ± 0.004 at the intra- and inter-population level, respectively.

### Microsatellites

Fourteen out of sixteen genotyped microsatellite loci were found polymorphic in at least one of the three sub-populations with a mean of four alleles by locus (Supplementary Table S2). Micro-Checker suggested the presence of null allele at the MCM139 locus. This locus was discarded from further analyses. Seven, two and three microsatellite loci deviated from HWE in Montes Forest, Mount Tonneri and Mount Lerno sub-populations, respectively (Supplementary Table S3). No locus deviated from HWE in all of the three sub-populations. The mean allelic richness across loci was higher (ANOVA: *P* < 0.01) in Montes Forest sub-population (4.17) when compared to Mount Tonneri (2.71) and Mount Lerno (2.54), indicating a higher genetic variability in this sub-population.

F_IS_ estimates were not statistically significant neither at the global nor at sub-population levels, except for the Montes Forest sub-population (Table 3).

**Table 3:** Estimates of microsatellite diversity indices for the three Sardinian mouflon sub-populations.

Sub-population	N	Allelic richness	Ho ± S.D	He ± S.D	F_IS_
Mount Lerno	15	2.54^a^	0.41 ± 0.31	0.43 ± 0.23	− 0.041
Mount Tonneri	21	2.71^a^	0.41 ± 0.27	0.38 ± 0.21	− 0.062
Montes Forest	18	4.17^b^	0.45 ± 0.26	0.49 ± 0.27	0.108*
Total	54	4.49^b^	0.42 ± 0.22	0.57 ± 0.14	0.037

*N* number of individuals per population, *Ho* observed heterozygosity (mean over loci), *He* expected heterozygosity (mean over loci), *F*_*IS*_ inbreeding coefficient calculated according to Weir & Cockerham (1984); significance was tested by 1000 bootstraps; *Statistically significant. Different superscript letters indicate statistical differences between sub-populations (ANOVA: *P* < 0.01).

The F_ST_ values were high both across the three sub-populations (F_ST_ = 0.30; 0.218–0.399; *P* < 0.01) and for pairwise comparisons, revealing a strong genetic differentiation. The highest genetic distance was recorded between Mount Lerno and Mount Tonneri (F_ST_ = 0.33; 0.174–0.461; *P* < 0.01), whereas the Montes Forest sub-population was found to be almost equally differentiated from the other two (vs Mount Lerno: F_ST_ = 0.27; 0.184–0.390; *P* < 0.01 and vs Mount Tonneri: F_ST_ = 0.27; 0.190–0.384; *P* < 0.01).

The AMOVA indicated that the within population variation accounted for 0.66% of total variation, whereas differences among populations contributed for 29.57% of the total variation (Table 2). In addition, most of the variance (69.77%) was partitioned within individuals reflecting a high genetic variation at the individual level.

**Figure 3:**
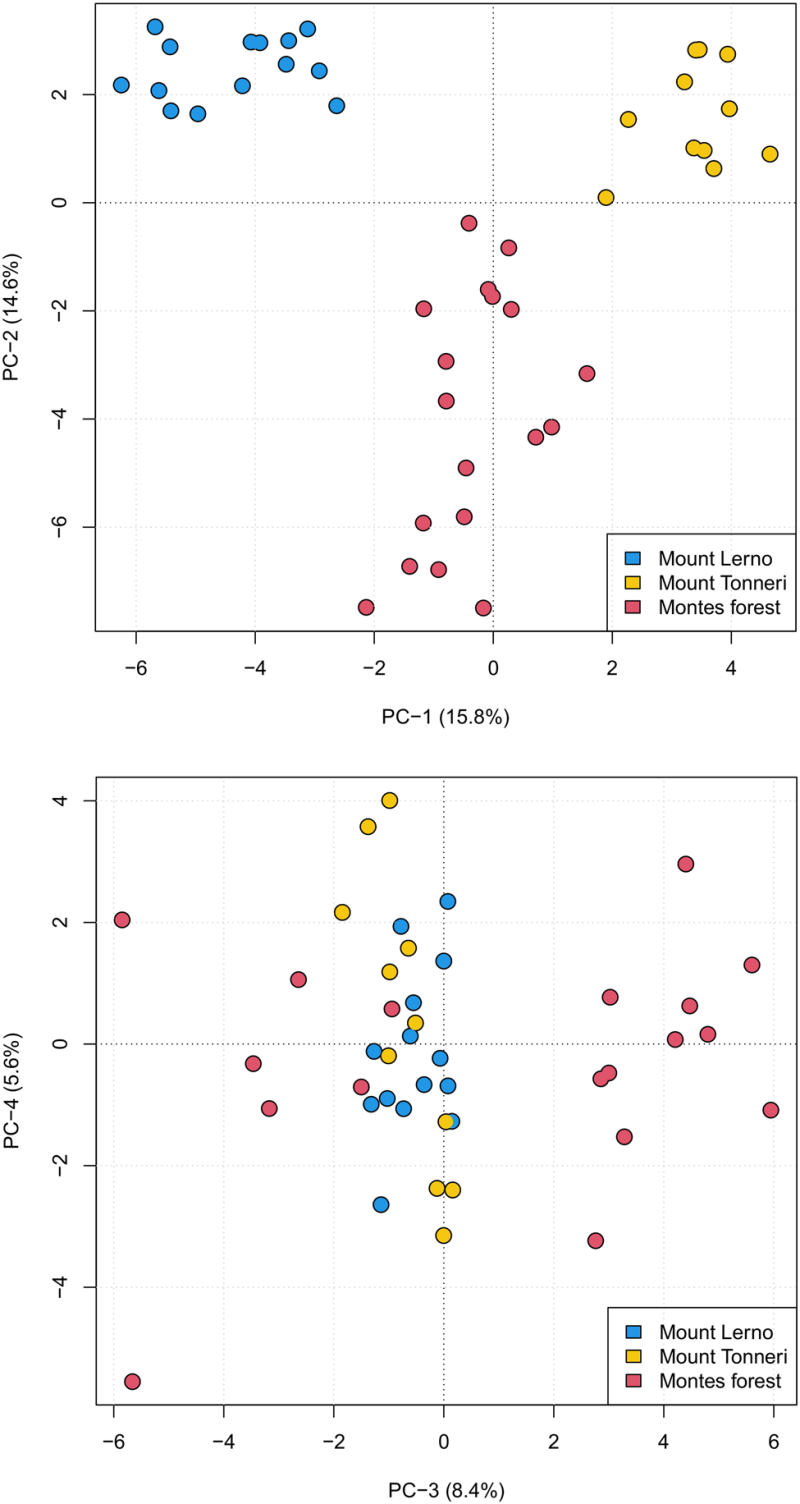
Principal component analysis of the three Sardinian mouflon sub-populations based on 13 microsatellite markers. The percentage values within brackets refer to the proportion of variance explained by each of the displayed principal components.

In the PCA (Fig. 3), the scree plot identified the first three principal components (PC) as the most informative (Supplementary Fig. S1). The first two PC combined accounted for 30.4% of the total variance and identified three distinct groups corresponding to the three sampling sites of Montes Forest, Mount Lerno and Mount Tonneri. One individual from the Mount Tonneri sub-population appeared in an intermediate position between the Mount Tonneri and Montes Forest groups. PC3 accounted for 8.4% of the variance and split the Montes Forest sub-population in two groups.

Since gene flow and inbreeding are known to affect allele frequencies, we assessed migration rate (Table 4) and relatedness. Data showed a low migration rate among sub-populations with the highest gene flow detected between Mount Tonneri and Montes Forest (2.1% migrants/generation). Lower values were found between Montes Forest and Mount Lerno (mean 1.8%), and between Mount Tonneri and Mount Lerno (mean 1.6%). Most of the individuals in each sub-population derived from their source population (Mount Lerno 96.23%; Mount Tonneri 96.58%; Montes Forest 96.19%).

**Table 4:** Means ± SD of posterior distributions of the migration rate into each sub-population.

	Mount Lerno	Mount Tonneri	Montes Forest
Mount Lerno	0.9623 ± 0.0238	0.0182 ± 0.0172	0.0186 ± 0.0177
Mount Tonneri	0.0141 ± 0.0137	0.9658 ± 0.0215	0.0201 ± 0.0172
Montes Forest	0.0179 ± 0.0170	0.0201 ± 0.0184	0.9619 ± 0.0243

The sub-populations into which individuals are migrating are listed in the rows, while the sources of the migrants are listed in the columns. Values along the diagonal represent the proportion of individuals from the source sub-populations each generation.

The median (± SD) values of the r frequency distributions inferred by the relatedness analysis were 0.054 (± 0.258) for Montes Forest, 0.351 (± 0.175) for Mount Lerno and 0.264 (± 0.227) for Mount Tonneri sub-populations.

The mean relatedness between sub-populations was 0.13, 0.33 and 0.37 for Montes Forest, Mount Tonneri and Mount Lerno, respectively (ANOVA: *P* < 0.01) (Supplementary Table S4). A multivariate analysis of pairwise relatedness distances identified two familial groups within the Montes Forest sub-population. No sub-structure was observed at the mtDNA level since the three haplotypes (Hpts 1–3) were found equally distributed across sub-populations (Supplementary Fig. S2).

**Figure 4:**
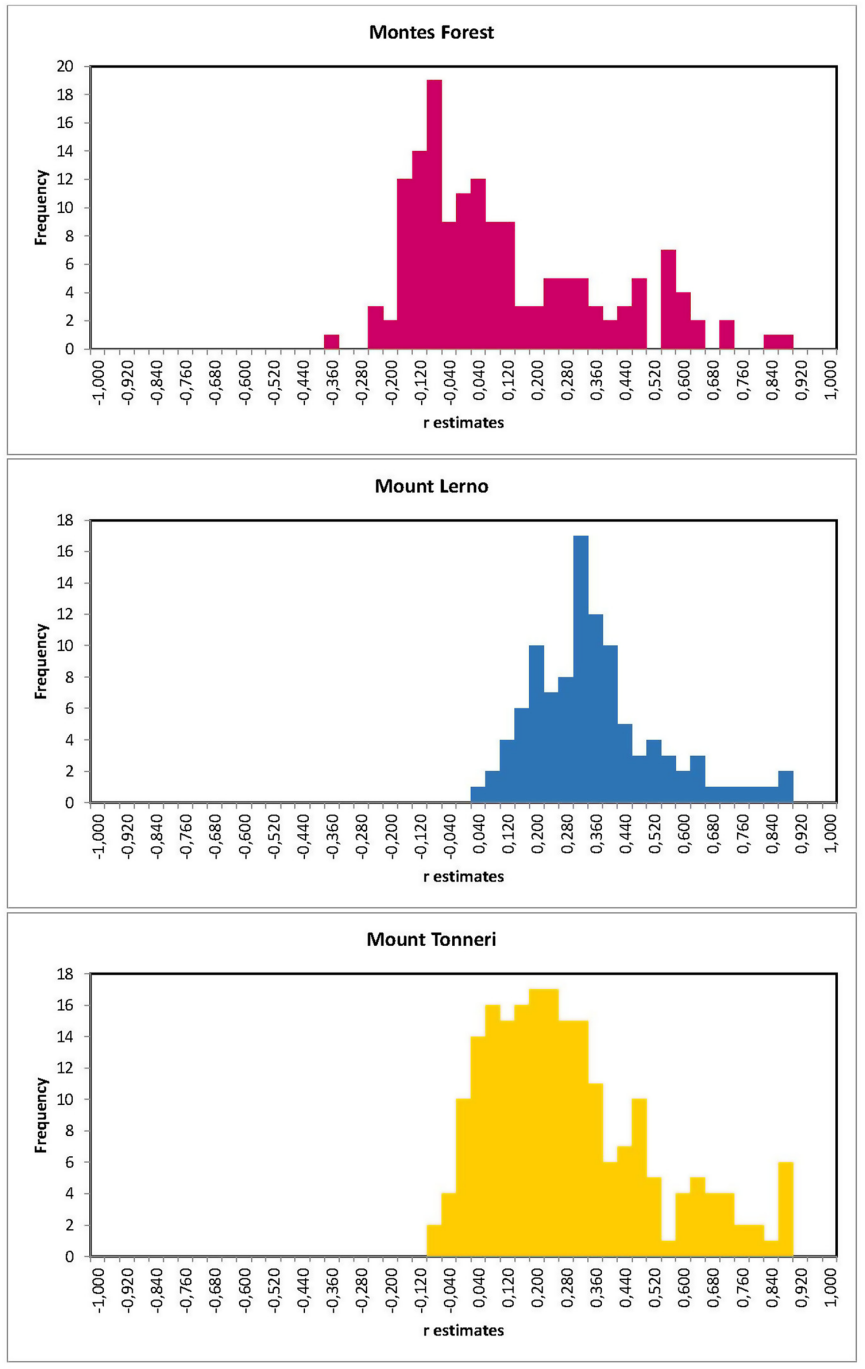
Distribution of pairwise relatedness coefficients showing the presence of highly related individuals in each of the three Sardinian mouflon sub-populations.

As expected, the presence of highly related individuals, either full siblings or parent-offsprings, was observed (r > 0.5; Fig. 4) with no statistical difference among sub-populations (χ^2^ test: *P* = 0.245). The proportion of r values >  + 0.5 was 13.1%, 19.2% and 19.6% in Montes Forest, Mount Tonneri and Mount Lerno sub-populations, respectively. One individual sourced from Mount Tonneri recorded the highest r estimates of 0.49 and 0.51 with one individual from Montes Forest and one from Mount Tonneri, respectively.

A strong signature of genetic bottleneck was detected in the Mount Lerno sub-population as indicated by the shifted mode curve resulting from the allelic frequencies distribution. Moreover, the one-tailed Wilcoxon test evidenced a significant heterozygosity excess for all the three mutation models (IAM, TPM and SMM; *P* < 0.05) (Table 5).

**Table 5:** Level of heterozygosity excess calculated under different mutation models in the three Sardinian mouflon sub-populations.

Sub-population	Mutation model	He	Hee	Wilcoxon sign rank test (P)	Mode-shift
Mount Lerno	IAM	9	5.45	0.00342*	SM
	SMM	9	6.09	0.03369*	
	TPM	9	6.18	0.03369*	
Mount Tonneri	IAM	9	6.43	0.05712	NLS
	SMM	7	7.12	0.47302	
	TPM	8	7.06	0.41968	
Montes Forest	IAM	8	7.09	0.08386	NLS
	SMM	6	7.49	0.79286	
	TPM	6	7.51	0.75134	

*He* observed number of loci with heterozygosity excess, *Hee* expected number of loci with heterozygosity excess, *IAM* infinite allele model, *SMM* stepwise mutation model, *TPM* two-phase model; *Significant difference between the observed and expected values for heterozygosity excess *p* < 0.05; *SM* shifted mode, *NLS* normal L-shaped.

Our results showed that the effective population size (*Ne*) was not affected by allele frequencies lower than 0.02. The Montes Forest sub-population showed the highest *Ne* mean value (2.8) compared to Mount Tonneri (2.4) and Mount Lerno (2.2) (Table 6). The analysis of the 95% CIs differences evidenced overlapping ranges between all sub-populations with the narrowest ranges in Montes Forest (CIs = 2.3–4.0) and Mount Tonneri (CIs = 1.7–3.3) sub-populations with respect to the Mount Lerno one (CIs = 1.4–5.3).

**Table 6:** Effective population size (*Ne*) of the three Sardinian mouflon sub-populations at different P_crit_ thresholds using the Linkage Disequilibrium method implemented in NeExtimator 2.

	n	Pcrit			
		0.050	0.020	0.010	0+
Mount Lerno	15	2.1 (1.3–4.4)	2.2 (1.4–5.3)	2.2 (1.4–5.3)	2.2 (1.4–5.3)
Mount Tonneri	21	2.2 (1.5–3.2)	2.4 (1.7–3.3)	2.4 (1.7–3.3)	2.4 (1.7–3.3)
Montes Forest	18	2.1 (1.7–2.5)	2.8 (2.3–4.0)	2.8 (2.3–4.0)	2.8 (2.3–4.0)

Parametric 95% Confidence Intervals (CIs) are indicated in brackets. N = n° of samples; P_crit_ = minimum allele frequency cut off.

## Discussion

Remnant island populations often represent unique genetic reservoirs due to their distinctiveness and should be always prioritised for conservation^31,32^. To this end, knowledge on their spatial distribution, population structure and connectivity are key to draw informed conservation plans^33–35^. We analysed 14 highly polymorphic microsatellite loci and D-loop sequences to describe the diversity and structure of the current Sardinian mouflon autochthonous population, providing the first evidence of a clear genetic structuring.

### Mitochondrial DNA

We detected a total of five mtDNA haplotypes, including Hpt-1 that has been described as the oldest haplotype within the HPG-B^8^. Among the other four, three were novel (Hpts 2, 3 and 4) while Hpt-5, found in the Mount Lerno population, was previously detected in the Comisana domestic sheep breed^30^ but never recorded in mouflon. Noticeably, none of the haplotypes detected within Montes Forest and Mount Tonneri sub-populations were found to be shared with domestic sheep. We hypothesize that during the subsequent steps of the translocation process from Mount Albo to Mount Lerno, passing through Capo Figari and the Asinara Island, contact with domestic breeds could have occurred.

Each sampling site showed a unique haplotype composition, suggesting a minimal matrilineal gene flow, in accordance with the high genetic differentiation detected among populations (F_ST_ = 0.79).

To position the Sardinian mtDNA haplotypes within a broader evolutionary context, we performed a network analysis including additional sequences of mouflon from Europe and Asia highlighting the presence of two clear phylogeographic clusters, including haplotypes of Asian and European origin, respectively (Fig. 2). Within the European cluster both Sardinian and Corsican mouflon showed private haplotypes, whereas two of the three European mouflon sequences grouped with two Asian mouflons in the same haplotype, as previously reported^5,8^. We speculate that the European and Asian mouflon individuals carrying this haplotype are likely to be mouflon rams *x* sheep ewe hybrids erroneously classified as pure mouflons based on morphological traits.

The central position of Hpt-1 in the network suggests its ancestral role in the phylogeny of *Ovis*. We found this haplotype in the Montes Forest sub-population, which supports it as the historical memory of the wild pool introduced in Sardinia by the first settlers during the Neolithic. However, additional data on Corsican mouflon are necessary to gain a complete view on the arrival of mouflon into the islands of the central Mediterranean area.

Our results highlighted the genetic differences between Corsico-Sardinian and European mouflons also found in previous works^5,8^, although additional sampling of individuals sourced from different parts of mainland Europe is necessary to confirm this observation. Importantly, the current nomenclature still aggregates Sardinian, Corsican, and mainland European mouflon under the same systematic classification. In 1994 Cugnasse^36^ suggested to refer to mainland mouflon populations with the scientific name *O. g. musimon* × *Ovis sp*. to account for their possibly impaired genetic integrity. Based on our mitochondrial results we believe appropriate to discriminate the Corsico-Sardinian mouflon and the mainland European mouflon as two different sub-species. A similar approach was implemented in the case of the Anatolian (*O. g. anatolica*) and Cypriot (*O. g. ophion*) mouflons^5,8,37^.

### Microsatellites

In accordance with the mtDNA-based AMOVA, microsatellite analyses showed high F_ST_ values and the presence of private alleles (n = 12), probably due to a reduced gene flow. However, caution is required in interpreting these results due to the small sample sizes available^38^. Previous studies on microsatellite loci^39,40^ recommended to sample at least 20–25 individuals to reduce errors and increase accuracy. Nonetheless, the calculation of genetic differentiation indices is still informative despite low sample sizes, as is often the case when studying small, endangered populations.

The mitochondrial/microsatellites F_ST_ ratio was > 2, suggesting higher male-biased natal dispersal. This is expected, as mouflon rams are more prone to disperse during rutting phase, albeit mouflon adopt a polygynous reproductive system with philopatric males and females^22,41,42^.

High levels of relatedness were recorded in all sub-populations likely due to inbreeding (Fig. 4). The polygynous mating behaviour of mouflon might also justify these levels of relatedness, due to restricted dispersal and the consequent increased relatedness among potential mates^43,44^. Furthermore, high relatedness is favoured in small populations where the low number of individuals increases the probability of mating with close relatives.

The PCA and F_ST_ results showed a clear differentiation among the three sub-populations. The presence of two groups detected in Montes Forest sub-population both by PCA and multivariate analysis of relatedness (Fig. 3; Supplementary Fig. S2) was consistent with the polygynous mating system. This cryptic intra-population structure has certainly played a role in driving the significant F_IS_ value and the low mean-relatedness recorded in Montes Forest.

The low migration rate and low *Ne* estimates suggest negligible levels of gene flow among the three sub-populations. However, both the PCA and the relatedness analysis identified an individual from Mount Tonneri showing admixed ancestry with Montes Forest. As this individual presented the mitochondrial haplotype Hpt-4 that we detected exclusively in the Mount Tonneri sub-population, we infer the individual to be a crossbred of a Montes Forest ram migrant and a Mount Tonneri ewe. This evidence suggests that a weak connectivity seems to persist, despite the very low levels of migration rate observed and the presence of artificial barriers limiting individuals’ dispersion (Fig. 1). Conversely, Mount Tonneri and Mount Lerno sub-populations showed the lowest migration rate, the highest microsatellite-based F_ST_ value, and the presence of private mtDNA haplotypes, suggesting a high degree of isolation.

The low allelic richness found in Mount Lerno and Mount Tonneri sub-populations was mirrored by a very low effective population size. Founder effect and genetic drift due to population contraction are known to cause loss of alleles and decrease of genetic variability^45,46^. However, recent bottleneck events are more sensitive to loss of alleles than to heterozygosity since the number of alleles drops more rapidly than allele frequencies^47,48^. Yet, a bottleneck event was only observed in Mount Lerno and not in Mount Tonneri. Nonetheless, the bottleneck detection procedure we implemented is known to have low prediction power when the effective size of the reduced population is too small and a new mutation-drift equilibrium is established^49^. We argue that the observed population contractions might be due to the devastating effects of forest fires that occurred in the past 20 years in the Mount Tonneri area (http.//www.regione.sardegna.it).

The low genetic variability currently observed in the Sardinian mouflon population might originate from the fragmentation of an original gene pool followed by long-term isolation. Originally, mouflon was widespread across Sardinia (Fig. 1) but human activities such as intensive agriculture, hunting, and high-density traffic roads, gradually lead to habitat erosion, dramatically reducing size and distribution of the population. The few areas that were only marginally affected are located in the Central-Eastern part of Sardinia, including Montes Forest and Mount Tonneri, due to their harsh orography. The genetic differentiation between Montes Forest and Mount Tonneri sub-populations could be the consequence of the landscape alterations (mainly due to a road expansion campaign started ~ 40 years ago), which may have led to the genetic depletion of the original gene pool by hindering gene exchange, and finally determining their genetic isolation. Similar situations were reported for the Californian desert bighorn sheep^50^ and other Sardinian macrofauna species such as wild boar and hare^51,52^.

## Conclusions

We investigated the diversity and population structure of three Sardinian mouflon sub-populations. Particularly, we highlight the genetic distinctiveness and degree of isolation of the Montes Forest and Mount Tonneri sub-populations. We argue that these sub-populations should be considered as separate management units and preserved from contacts with genetically uncharacterized and known mouflon *x* sheep hybrid populations, such as the Mount Lerno population. Hybridization of wild populations with their domestic counterparts can lead to the loss of wildtype genetic integrity, outbreeding depression, and loss of adaptive features^53–55^. Morphological features are rarely informative to discriminate hybrids from pure mouflons especially when several events of backcrosses occurred^55^. Conversely, molecular investigations have the power to identify mixed ancestry components even with reduced panels of selected ancestry informative markers and are the only diagnostic tools able to effectively identify hybrids before carrying out restocking actions^26,55^. Our study prompts for initiatives to restore the habitat connectivity between Montes Forest and Mount Tonneri and promotes controlled inter-population exchange in order to improve the overall genetic diversity and counteract the effects of population fragmentation^56,57^. The connectivity disruption effect has been more severe in the Mount Tonneri sub-population as evidenced by the reduction in population size, *Ne* and allelic richness, probably due to forest fires. Actions as the implementation of biological corridors such as overpasses would allow continuous exchange of individuals among sub-populations, thus reducing the local extinction rate^58^. Higher gene flow leads to higher heterozygosity, which is often associated with increased fitness (heterozygote advantage), while the risk of outbreeding depression is overall minimal when the populations involved inhabit the same habitat^59^. This is the case for Montes Forest and Mount Tonneri sub-populations that originated in the last century from the same original gene pool, are quite close to each other and host identical habitats. The present study represents a first important step towards a deeper understanding of the genetic peculiarities of the native mouflon populations living in Sardinia. Our results highlight the deleterious effects on genetic diversity of habitat changes and fragmentation of the original gene pool. Given the key role of the Sardinian mouflon in the recolonization of continental Europe, such a knowledge is an indispensable prerequisite for the protection of its genetic identity, jeopardised by uncontrolled interaction with domestic sheep breeds.

## Methods

All the animal procedures were performed in compliance with the ARRIVE guidelines and in strict accordance with the guidelines of the Ethics Committee of Sassari University, Italy, which also approved this study.

### Sample collection

We collected either peripheral blood (captured mouflon) or muscular tissue samples (dead individuals) from 54 Sardinian mouflon from three distinct sites of the island during monitoring actions carried out from 2010 to 2016. A total of 39 samples (18 from Montes Forest, Orgosolo, and 21 from Mount Tonneri, Seui) were collected in the historical mouflon range. Other 15 samples were sampled from Mount Lerno (Pattada) site, where mouflons were reintroduced 30 years ago by three subsequent transfers, sourcing from Mount Albo and passing through Capo Figari and the Asinara Island (Fig. 1). Unfortunately, the collection of specimens from Mount Albo (Lula), the third historical site left on the island, was not successful due to the small population density in that area. Currently, these three sampling areas are partially isolated by recently built roads and dikes (Fig. 1) and no chance of exchange among populations exists.

### DNA extraction, amplification and sequencing

Genomic DNA was extracted from blood and muscle using the GenElute Blood Genomic DNA kit (Sigma-Aldrich, Darmstadt, Germany) and the NucleoSpin Tissue XS Kit (Macherey-Nagel), respectively. Procedures were carried out according to the manufacturer's protocols. Sample quality and DNA concentration were determined via spectrophotometry using a ND-8000 (NanoDrop Technologies, Thermo Fisher Scientific Inc., Wilmington, DE). To ensure the sequence accuracy, DNA was sequenced from PCR replicates. DNA samples were used to assay 16 microsatellite loci length and the mitochondrial D-loop region polymorphisms in order to genetically characterize each group and to clarify the genetic relationships among them.

### Mitochondrial DNA

The primer pair CR1-CR2 (Sanna et al., 2015) was used to amplify the mtDNA D-loop fragment. A standard 50 μL PCR mixture was used, including 200 ng DNA template, 2.5 mM MgCl_2_, 0.2 0 mM each dNTP, 0.20 μM each primer, 0.02 mM BSA, 1 × PCR buffer and 2 units Taq DNA Polymerase (Sigma-Aldrich), according to Mereu et al.^32^. PCR amplifications were performed in a Gradient Thermocycler (Eppendorf) by an initial denaturation of 95 °C for 3 min, followed by 30 cycles of 95 °C for 50 s, 60 °C for 30 s, and 72 °C for 1 min.

PCR products were sequenced using the same primers on an ABI 3130 Genetic Analyzer (Applied Biosystem). Sequencing reactions were carried out following the manufacturer's recommendations (BigDye Terminator 3.1 Cycle Sequencing Kit—Applied Biosystem) and purified through the SigmaSpin Post—Reaction Clean—UP Columns (Sigma-Aldrich).

Raw sequencing data were processed by means of the KB base-calling algorithm implemented in the Sequencing Analysis Software 5.3.1 (Applied Biosystem).

### Haplotypes and population structure analysis

Sequence alignment and data formatting, including trimming to a core length of 439 bp and resolving the ambiguities (N) in base-calling, were performed using BioEdit^60^.

The amount of genetic variation among the three sub-populations, including the number of polymorphic sites (S) and haplotypes (h), the haplotype diversity (Hd) and nucleotide diversity (π), were estimated using DnaSP 6.10.03^61^.

The analysis of molecular variance (AMOVA) and the calculation of the coefficient of differentiation (F_ST_) were carried out using Arlequin 3.5^62^.

Genetic relationships among haplotypes were investigated by a median joining network (MJN) on the nucleotide sequence matrix of 91 D-loop sequences. To explore the evolutionary relationships between the Sardinian mouflon and other *O. gmelini* species, the dataset was combined with a total of 34 homologous sequences of mouflons from Cyprus (n = 3), Corsica (n = 2), mainland Europe (n = 3), Anatolia (n = 16) and Asia (n = 10). These sequences were a representative sub-sample of all the haplotypes deposited in GenBank and were selected, one for each haplotype, on the basis of similarity detected at the BLAST analysis. Three *O. vignei* sequences were also included as outgroup (Supplementary Table S5). The MJN was constructed in PopART 1.750 [http://popart.otago.ac.nz].

The pairwise genetic distances were estimated with the software MEGA 7.0.14^63^ using 10,000 bootstrap replicates. The Tamura-Nei (TN93)^64^ model of nucleotide substitution with uniform evolutionary rates among sites was chosen to estimate genetic distance between haplotypes on the basis of the lower Bayesian information criterion (BIC) score.

### Microsatellites

We genotyped 54 samples at 14 microsatellites designed for domestic sheep (*O. aries*) and cattle (*Bos taurus*) obtained from public databases (NCBI genome database, http://www.ncbi.nlm.nih.gov; ARKdb database, http://www.thearkdb.org). Markers were amplified by PCR in 25 µl reaction mix containing: 10 μl of DNA (1 ng/μl) as a template, 1X PCR Buffer, 1.5 mM of each dNTP, 0.5 μM of each primer, 1 unit of Taq Polymerase and 1.5 mM MgCl_2_. PCR amplifications were performed in a Gradient Thermocycler (Eppendorf) by an initial denaturation of 95 °C for 2 min, followed by 35 cycles of 94 °C for 30 s, 48–58 °C for 1 min (Supplementary Table S6) and 72 °C for 1 min, followed by a final step of 72 °C for 5 min.

Microsatellite products were analysed on an ABI PRISM 3100 DNA Analyzer (Applied Biosystems) and data processed using GeneScan 3.1 and Genotyper 2.5^65^. Identification of possible genotyping errors due to null alleles, short allele dominance and typographic errors, were tested using Micro-Checker software^66^ (http://www.microchecker.hull.ac.uk). Allelic richness and F_IS_ values were calculated using Fstat 2.9.3^67^. Differences between populations in allelic richness were tested using the Fisher exact test method, after assessing for normality of the data and variance homogeneity (Minitab 17 statistical software, Minitab, Inc. State College, PA), while F_IS_ significance was tested by 95% confidence interval with 1000 bootstrap replicates by Fstat 2.9.3. Allele frequency, gene diversity (expected heterozygosity—He, Nei 1973), observed heterozygosity (Ho) and Hardy–Weinberg equilibrium (HWE) deviation analyses by population and locus were calculated using the Fisher exact test^68^ in a contingency table of arbitrary size, as implemented in Arlequin 3.5^62^. The exact *P*-values were obtained using a Markov Chain Monte Carlo (MCMC) simulation with 10,000 dememorization steps, 500 batches, and 5000 iterations.

Genetic differentiation (F_ST_) used as inter-population indices to assess the relationships among the three mouflons groups was calculated by permuting the genotypes or individuals between the sub-populations. The significance of the genetic distances was tested by Weir & Cockerham^69^ as implemented in Arlequin 3.5 (10,000 permutations).

Population genetic structure was investigated using the analysis of molecular variance (AMOVA) by Arlequin 3.5.

A principal component analysis (PCA) was performed to investigate the ordinal relationships between populations and individuals, using the ade4 1.7-16 package^70^ of the R software^71^.

Recent migration rates among sub-populations were assessed using a Bayesian Markov Chain Monte Carlo (MCMC) analysis implemented in BayesAss 1.3^72^ which allows estimation of the rate and direction of recent dispersal. The MCMC method was run for 10,000,000 iterations. Delta values (i.e., maximum parameter change per iteration) were left as default^73^.

Pairwise relatedness (r) between individuals within populations based on the method of Lynch and Ritland^74^ with the 2x option was estimated using GenAlEx 6.501^75^ to detect related individuals within the same sub-population. This method assumes values from − 1 (completely unrelated individuals) to 1 (identical twins). To calculate pairwise relatedness, we used the allele frequency of the total population of assembled genotypes with 9999 permutations and r frequencies were plotted to evaluate its distribution. Distribution factors such as median and mean were compared by χ^2^ test and ANOVA test respectively, using Minitab 17. Relatedness estimates higher than 0.5 observed in each population were compared in the whole sample by means of Pearson χ^2^ test (Minitab 17). To assess the presence of sub-structures within sub-populations a matrix of pairwise relatedness distances between individuals was computed by Genalex 6.501 and a multivariate analysis was performed using the Cluster Variables function implemented in Minitab 17. The results were graphically expressed by a UPGMA tree (Minitab 17).

Bottleneck events were detected from allele frequency data by the BOTTLENECK software^76^ assuming that microsatellite mutation followed the Infinite Allele Model (IAM), Stepwise Mutation Model (SMM) or Two-Phase Model (TPM)^77^. In TPM we used 95% single-step mutational events at 12% variance. To determine the significance in the heterozygosity excess we performed the one-tailed Wilcoxon test. A mode-shift test was carried out to detect a distortion on the expected L-shaped distribution of allele frequency and to determine whether the population is under mutation drift equilibrium (shaped curve) or not (mode shift)^78^.

We estimated the effective population size using the Linkage disequilibrium method implemented in NeEstimator 2^79^. This method has been proven relatively robust in assessing the effective population size (*Ne*) using microsatellite data^80^. As rare alleles could result in biased *Ne*, we calculated three estimates for *Ne* by excluding allele frequencies of less than (Pcrit) 0.05, 0.02, or 0.01. The 95% CI was estimated by the parametric method^81^.

## Supplementary Information

Supplementary Figure S1.

Supplementary Figure S2.

Supplementary Table S1.

Supplementary Table S2.

Supplementary Table S3.

Supplementary Table S4.

Supplementary Table S5.

Supplementary Table S6.
