# Supplementary material for: Genetic characterization and implications for conservation of the last autochthonous Mouflon population in Europe
Source: Sci Rep. 2021 Jul 19;11:14729. doi: 10.1038/s41598-021-94134-3 (PMC8289818; doi:10.1038/s41598-021-94134-3)

GENETIC CHARACTERIZATION AND IMPLICATIONS FOR CONSERVATION OF THE LAST AUTOCHTHONOUS MOUFLON POPULATION IN EUROPE

Valentina Satta, Paolo Mereu, Mario Barbato, Monica Pirastru, Giovanni Bassu, Laura Manca, Salvatore Naitana, Giovanni Giuseppe Leoni.

Supplementary Fig. S2. Dendrogram of relatedness pairwise distances among individuals within Montes Forest sub-population. The circles represent the mtDNA haplotypes; green circles: Hpt-1, blue circles: Hpt-2, red circles: Hpt-3.

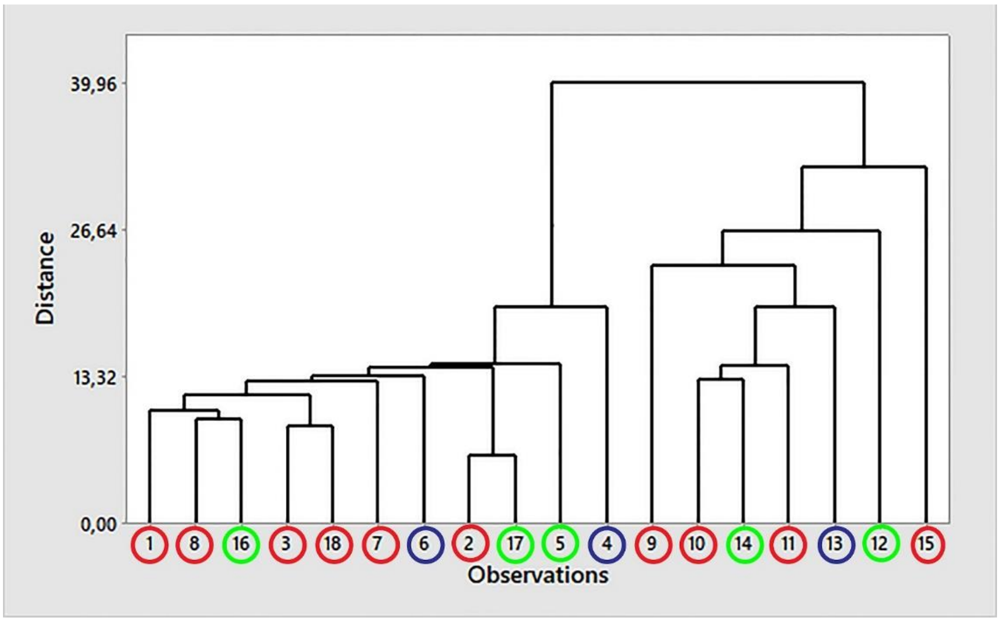

Supplement: Supplementary file 2 — Supplementary Figure S2. [file 41598_2021_94134_MOESM2_ESM.pdf]
