# Supplementary material for: Genetic characterization and implications for conservation of the last autochthonous Mouflon population in Europe
Source: Sci Rep. 2021 Jul 19;11:14729. doi: 10.1038/s41598-021-94134-3 (PMC8289818; doi:10.1038/s41598-021-94134-3)
Supplement: Supplementary file 3 — Supplementary Table S1. [file 41598_2021_94134_MOESM3_ESM.pdf]

GENETIC CHARACTERIZATION AND IMPLICATIONS FOR CONSERVATION OF THE LAST AUTOCHTHONOUS MOUFLON POPULATION IN EUROPE

Valentina Satta, Paolo Mereu, Mario Barbato, Monica Pirastru, Giovanni Bassu, Laura Manca, Salvatore Naitana, Giovanni Giuseppe Leoni.

Supplementary Table S1. Estimates of evolutionary divergence calculated under the TN-93 model among mtDNA D-loop haplotypes detected in the 54 samples from the three Sardinian mouflon sub-populations. Genetic distances are shown below the diagonal and standard deviations above the diagonal.

	**Hpt-3**	**Hpt-2**	**Hpt-1**	**Hpt-4**	**Hpt-5**
**Hpt-3**		0.00298	0.00298	0.00440	0.00497
**Hpt-2**	0.00420		0.00242	0.00433	0.00450
**Hpt-1**	0.00419	0.00683		0.00441	0.00465
**Hpt-4**	0.00843	0.01059	0.01062		0.00437
**Hpt-5**	0.01066	0.00992	0.01002	0.00986	
