# Supplementary material for: Genetic characterization and implications for conservation of the last autochthonous Mouflon population in Europe
Source: Sci Rep. 2021 Jul 19;11:14729. doi: 10.1038/s41598-021-94134-3 (PMC8289818; doi:10.1038/s41598-021-94134-3)
Supplement: Supplementary file 4 — Supplementary Table S2. [file 41598_2021_94134_MOESM4_ESM.pdf]

GENETIC CHARACTERIZATION AND IMPLICATIONS FOR CONSERVATION OF THE LAST AUTOCHTHONOUS MOUFLON POPULATION IN EUROPE

Valentina Satta, Paolo Mereu, Mario Barbato, Monica Pirastru, Giovanni Bassu, Laura Manca, Salvatore Naitana, Giovanni Giuseppe Leoni.

Supplementary Table S2. Allelic frequencies in the sixteen microsatellite loci genotyped into the three Sardinian mouflon populations

Marker	Alleles	Mount Lerno (n=15)	Mount Tonneri (n=21)	Montes Forest (n=18)
**BM1824**	1	0.17	0	0.28
	2	0.40	0.98	0.64
	3	0.33	0.02	0.08
	4	0.10	0	0
**MC218**	1	0	0	0.03
	2	0.83	0.05	0.17
	3	0	0.74	0
	4	0.17	0.21	0.80
**BM6041**	1	0	0.14	0.05
	2	1.00	0.76	0.67
	3	0	0	0.03
	4	0	0	0.22
	5	0	0.10	0.03
**MCM150**	1	0	0.26	0
	2	0.50	0.74	0.97
	3	0.50	0	0.03
**MC138**	1	0.27	0.07	0.94
	2	0.73	0.93	0.06
**MNS5**	1	0.43	0.53	0.58
	2	0	0	0.31
	3	0.57	0.14	0.08
	4	0	0	0.03
	5	0	0.33	0
**MCMA26**	1	0	0	0.03
	2	0	0.14	0
	3	0	0.72	0.31
	4	0	0.10	0
	5	0	0.02	0.08
	6	0	0.02	0.19
	7	0.27	0	0.11
	8	0.40	0	0.28
	9	0.33	0	0
**MCM14**	1	0	0	0.14
	2	0	0	0.03
	3	0	0	0.22
	4	0	0	0.14
	5	0	0	0.17
	6	0	0.05	0
	7	0.17	0.05	0
	8	0.47	0.57	0.22
	9	0.37	0.33	0.08
**BM1714**	1	0.30	0	0
	2	0	0	0.06
	3	0.53	0.62	0.19
	4	0.17	0.07	0.53
	5	0	0.31	0.22

**MCM139**	1	0	0.00	0
	2	0.33	1	1
	3	0.60	0	0
	4	0.07	0	0
	5	0	0.	0
**MCMA1**	1	0	0	0.11
	2	0.57	0	0.16
	3	0	0	0.17
	4	0	0	0.06
	5	0.30	0.69	0.28
	6	0	0.31	0
	7	0	0	0.14
	8	0.13	0	0.05
	9	0	0	0.03
**BM4006**	1	0.67	0	0.03
	2	0	0	0.14
	3	0.033	0.02	0.11
	4	0.30	0.98	0.55
	5	0	0	0.17
**MCM203**	1	0.13	0.17	0
	2	0.77	0.24	0.61
	3	0	0.59	0.39
	4	0.10	0	0
**BM827**	1	1	0.57	0.69
	2	0	0	0.19
	3	0	0.43	0.03
	4	0	0	0.06
	5	0	0	0.03
