# Supplementary material for: Genetic characterization and implications for conservation of the last autochthonous Mouflon population in Europe
Source: Sci Rep. 2021 Jul 19;11:14729. doi: 10.1038/s41598-021-94134-3 (PMC8289818; doi:10.1038/s41598-021-94134-3)
Supplement: Supplementary file 5 — Supplementary Table S3. [file 41598_2021_94134_MOESM5_ESM.pdf]

GENETIC CHARACTERIZATION AND IMPLICATIONS FOR CONSERVATION OF THE LAST AUTOCHTHONOUS MOUFLON POPULATION IN EUROPE

Valentina Satta, Paolo Mereu, Mario Barbato, Monica Pirastru, Giovanni Bassu, Laura Manca, Salvatore Naitana, Giovanni Giuseppe Leoni.

Supplementary Table S3. Observed (Ho) and expected (He) heterozygosity and deviation from Hardy Weinberg equilibrium estimated for the 14 polymorphic microsatellite loci genotyped into the three Sardinian mouflon sub-populations.

X = monomorphic locus into a single sub-population. * Indicates statistical deviation from HW equilibrium (χ^2 test: P<0.05; Guo and Thompson (1992)).

	Mount Lerno (n=15)				Mount Tonneri (n=21)				Montes Forest (n=18)				Total (n=54)			
Locus	Ho	He.	P-value	S.D.	Ho	He.	P-value	S.D.	Ho	He.	P-value	S.D.	Ho	He.	P-value	S.D
BM1824	0.73333	0.70805	0.54798	0.00055	0.09524	0.09292	1.00000	0.00000	0.55556	0.52222	0.01666*	0.00013	0.42593	0.48062	0.33904	0.00045
MC218	0.20000	0.18621	1.00000	0.00000	0.61905	0.49361	0.43425	0.00053	0.33333	0.33175	0.20654	0.00036	0.40741	0.66926	0.00009*	0.00001
BM6041	X				0.47619	0.45993	0.40436	0.00047	0.61111	0.53968	1.00000	0.00000	0.40741	0.40897	0.53665	0.00041
MC150	0.66667	0.51494	0.32481	0.00048	0.57143	0.41812	0.13037	0.00034	0.11111	0.10794	1.00000	0.00000	0.44444	0.44237	0.14242	0.00032
MC138	0.26667	0.40460	0.22594	0.00042	0.14286	0.13589	1.00000	0.00000	0.11111	0.10794	1.00000	0.00000	0.16667	0.49065	0.00000*	0.00000
MNS5	0.60000	0.48046	0.57771	0.00049	0.80952	0.63298	0.42188	0.00047	0.33333	0.61429	0.00055*	0.00002	0.59259	0.68311	0.00001*	0.00000
MCMA26	0.86667	0.65287	0.33029	0.00045	0.57143	0.50290	0.26501	0.00037	0.77778	0.79365	0.00079*	0.00003	0.72222	0.79145	0.00000*	0.00000
MCM14	0.73333	0.64138	0.65064	0.00050	0.66667	0.60511	0.84891	0.00034	0.72222	0.85079	0.01760*	0.00012	0.70370	0.75130	0.00314*	0.00006
BM1714	0.66667	0.65977	0.08432	0.00029	0.61905	0.52846	0.02587*	0.00015	0.72222	0.66508	0.28555	0.00043	0.66667	0.70111	0.07068	0.00025
MCM139	0.00000	0.60690	0.00000*	0.00000	X				X				0.00000	0.31568	0.00000*	0.00000
MCMA1	0.60000	0.59080	0.00203*	0.00004	0.61905	0.43786	0.11547	0.00032	0.77778	0.85238	0.00495*	0.00008	0.66667	0.73936	0.00000*	0.00000
BM4006	0.33333	0.48046	0.29719	0.00046	0.04762	0.04762	1.00000	0.00000	0.33333	0.64921	0.00220*	0.00004	0.22222	0.51869	0.00000*	0.00000
MCM203	0.06667	0.19080	0.03483*	0.00017	0.23810	0.57491	0.00108*	0.00003	0.55556	0.48889	0.64596	0.00047	0.29630	0.56940	0.00000*	0.00000
BM827	X				0.28571	0.50174	0.07608	0.00027	0.33333	0.48889	0.01651*	0.00014	0.22222	0.43337	0.00000*	0.00000
