# Supplementary material for: Genetic characterization and implications for conservation of the last autochthonous Mouflon population in Europe
Source: Sci Rep. 2021 Jul 19;11:14729. doi: 10.1038/s41598-021-94134-3 (PMC8289818; doi:10.1038/s41598-021-94134-3)
Supplement: Supplementary file 6 — Supplementary Table S4. [file 41598_2021_94134_MOESM6_ESM.pdf]

GENETIC CHARACTERIZATION AND IMPLICATIONS FOR CONSERVATION OF THE LAST AUTOCHTHONOUS MOUFLON POPULATION IN EUROPE

Valentina Satta, Paolo Mereu, Mario Barbato, Monica Pirastru, Giovanni Bassu, Laura Manca, Salvatore Naitana, Giovanni Giuseppe Leoni.

Supplementary Table S4. Mean relatedness in the three Sardinian mouflon sub-populations.

The superscripts indicate pairwise comparisons statistically different (ANOVA: P = 0.000).

Sub-Population	Mean	S.D.	95% C.I.
**Mount Lerno**	0.3747[a]	0.1741	0.3318; 0,4175
**Montes Forest**	0.1354[b]	0.2628	0,0991; 0,1717
**Mount Tonneri**	0.33077[c]	0.2271	0,2767; 0,3387
