# Supplementary material for: Genetic characterization and implications for conservation of the last autochthonous Mouflon population in Europe
Source: Sci Rep. 2021 Jul 19;11:14729. doi: 10.1038/s41598-021-94134-3 (PMC8289818; doi:10.1038/s41598-021-94134-3)
Supplement: Supplementary file 7 — Supplementary Table S5. [file 41598_2021_94134_MOESM7_ESM.pdf]

GENETIC CHARACTERIZATION AND IMPLICATIONS FOR CONSERVATION OF THE LAST AUTOCHTHONOUS MOUFLON POPULATION IN EUROPE

Valentina Satta, Paolo Mereu, Mario Barbato, Monica Pirastru, Giovanni Bassu, Laura Manca, Salvatore Naitana, Giovanni Giuseppe Leoni.

Supplementary Table S5. List of additional mtDNA D-loop sequences from *Ovis gmelini* individuals sampled in other geographical areas which were included in the Median Joining Network analysis.

#GB: GenBank Accession Number

	#GB	Species	Common name	Country
1	MT768232	*Ovis gmelini*	Asian mouflon	Missing information
2	MT768231	*Ovis gmelini*	Asian mouflon	Missing information
3	MT768222	*Ovis gmelini*	Asian mouflon	Missing information
4	MT768221	*Ovis gmelini*	Asian mouflon	Missing information
5	MT768196	*Ovis gmelini*	Asian mouflon	Missing information
6	MT768146	*Ovis gmelini*	Asian mouflon	Missing information
7	MT768074	*Ovis gmelini*	Asian mouflon	Missing information
8	MT768073	*Ovis gmelini*	Asian mouflon	Missing information
9	NC_026063	*Ovis gmelini*	Asian mouflon	Missing information
10	KF938360	*Ovis gmelini*	Asian mouflon	Kazakhstan
11	KF677292	*Ovis gmelini anatolica*	Anatolian mouflon	Turkey
12	KF677293	*Ovis gmelini anatolica*	Anatolian mouflon	Turkey
13	KF677291	*Ovis gmelini anatolica*	Anatolian mouflon	Turkey
14	KF677290	*Ovis gmelini anatolica*	Anatolian mouflon	Turkey
15	KF677289	*Ovis gmelini anatolica*	Anatolian mouflon	Turkey
16	KF677288	*Ovis gmelini anatolica*	Anatolian mouflon	Turkey
17	KF677287	*Ovis gmelini anatolica*	Anatolian mouflon	Turkey
18	KF677286	*Ovis gmelini anatolica*	Anatolian mouflon	Turkey
19	KF677285	*Ovis gmelini anatolica*	Anatolian mouflon	Turkey
20	KF677284	*Ovis gmelini anatolica*	Anatolian mouflon	Turkey
21	KF677277	*Ovis gmelini anatolica*	Anatolian mouflon	Turkey
22	KF677275	*Ovis gmelini anatolica*	Anatolian mouflon	Turkey
23	KF677271	*Ovis gmelini anatolica*	Anatolian mouflon	Turkey
24	KF677266	*Ovis gmelini anatolica*	Anatolian mouflon	Turkey
25	KF677265	*Ovis gmelini anatolica*	Anatolian mouflon	Turkey
26	KF677264	*Ovis gmelini anatolica*	Anatolian mouflon	Turkey
27	KR011779	*Ovis gmelini ophion*	Anatolian mouflon	Cyprus
28	KR011780	*Ovis gmelini ophion*	Anatolian mouflon	Cyprus
29	KF312238	*Ovis gmelini ophion*	Anatolian mouflon	Cyprus
30	HM236185	*Ovis gmelini musimon*	European mouflon	Germany
31	HM236184	*Ovis gmelini musimon*	European mouflon	Germany
32	AF039579	*Ovis gmelini musimon*	European mouflon	Germany
33	KR011782	*Ovis gmelini musimon*	Corsican mouflon	France, Corse
34	KR011781	*Ovis gmelini musimon*	Corsican mouflon	France, Corse
35	KF938361	*Ovis vignei*	Urial	Kazakhstan
36	NC_026064	*Ovis vignei*	Urial	Missing information
37	MT768097	*Ovis vignei*	Urial	Missing information
