# Supplementary material for: Genetic characterization and implications for conservation of the last autochthonous Mouflon population in Europe
Source: Sci Rep. 2021 Jul 19;11:14729. doi: 10.1038/s41598-021-94134-3 (PMC8289818; doi:10.1038/s41598-021-94134-3)
Supplement: Supplementary file 8 — Supplementary Table S6. [file 41598_2021_94134_MOESM8_ESM.pdf]

GENETIC CHARACTERIZATION AND IMPLICATIONS FOR CONSERVATION OF THE LAST AUTOCHTHONOUS MOUFLON POPULATION IN EUROPE

Valentina Satta, Paolo Mereu, Mario Barbato, Monica Pirastru, Giovanni Bassu, Laura Manca, Salvatore Naitana, Giovanni Giuseppe Leoni.

Supplementary Table S6 Nucleotide sequences and annealing temperatures of primer pairs used in microsatellite analyses of the three Sardinian mouflon sub-populations.

Ta = annealing temperature.

Marker	Stain	Primer sequences	Ta °C	Source
BM1824	Hex	F:GAGCAAGGTGTTTTTCCAATC<br>R:CATTCTCCAACTGCTTCCTTG	54	*Bos taurus*
BM4006	6-Fam	F:CAATGTGCATTATTTCCAAAGTG<br>R:AGAGATAACTCTTTCTTCTTGGAGG	53	*Bos taurus*
BM0827	Hex	F:GGGCTGGTCGTATGCTGAG<br>R:GTTGGACTTGCTGAAGTGAAC	56	*Bos taurus*
MCMO218	6-Fam	F:GATCCTAGCATCAGTCTCCAGATG<br>R:CACTAAAAGCTTATGAAAGTTCCAGC	57	*Ovis aries*
MCMA14	Hex	F:TGTTTCCTCTTCTCCAAATATC<br>R:GCCCTATTAAGCCAATATACAG	49	*Ovis aries*
MCM139	6-Fam	F:CTAGGGCTATAGAGGATGGTGTGT<br>R:TCGGACACAACTGAGCATGTG	55	*Ovis aries*
MCM138	Hex	F:AGAAATGCGTGTTTTCAAGGC<br>R:TTTAACTTCTTCATTTCCATTGTGTG	48	*Ovis aries*
INRA0013	6-Fam	F:GCACAGTGACCTCTCAATAAATGC<br>R:CCACTATTCTTGCCTGAAGAATCC	58	*Bos taurus*
MCMO150	Hex	F:AGGAAAATCTTCCGGAGCTAAAC<br>R:CCACTTGGAGTGAAAATGAGACA	56	*Ovis aries*
BM1862	6-Fam	F:AAGCAAAAAGGCTGATGGC<br>R:TTGCAGATACTGGCAAGTGG	56	*Bos taurus*
MCMA26	Hex	F:TCTCTGCTTTCCAGCCTTATTC<br>R:AGAGCTTTTAGGACAGCCACC	55	*Ovis aries*
MCM203	6-Fam	F:TACGCTAAGGGTTAAACAGGCC<br>R:CCAATGAGGACAATGATGCAC	55	*Ovis aries*
BM6041	Hex	F:GGCTGCTGCATGTCAGTG<br>R:GACTTGAGCTCCTCCAGGG	58	*Bos taurus*
MCMA01	6-Fam	F:CATTACAGCCTGTGTGAGTGTG<br>R:GATAGTTCTATCCAACCGTCCC	55	*Ovis aries*
BMS1714	Hex	F:TTTATCCCAAGAGGTTCCACC<br>R:AGGTGCTTGCAGTGAATCTG	55	*Bos taurus*
MNS5	6-Fam	F:ACAGCAGCAAAGACTCAGCA<br>R:GAAGAAGAAACATGATGGGCA	55	*Ovis aries*
